# Urinary metabolomics signature of animal and plant protein intake and its association with 24-h blood pressure: the African-PREDICT study

**DOI:** 10.1038/s41440-024-01767-8

**Published:** 2024-07-04

**Authors:** Michél Strauss-Kruger, Marlien Pieters, Tertia van Zyl, Ruan Kruger, Adriaan Jacobs, Esmé Jansen van Vuren, Roan Louw, Carina (C. M. C) Mels

**Affiliations:** 1 Hypertension in Africa Research Team (HART), North-West University, Potchefstroom, 2520 North-West Province South Africa; 2 SAMRC Extramural Unit for Hypertension and Cardiovascular Disease, Faculty of Health Sciences, North-West University, Potchefstroom, South Africa; 3 Centre of Excellence for Nutrition (CEN), North-West University, Potchefstroom, 2520 South Africa; 4 Human Metabolomics, North-West University, Potchefstroom, 2520 North-West Province South Africa

**Keywords:** Animal protein intake, Blood pressure, Metabolomics, Plant protein intake

## Abstract

The contrasting relationships of plant and animal protein intake with blood pressure (BP) may be partially attributed to the differential non-protein (e.g., saturated fat and fibre) and amino acid (AA) compositions. This study determined whether animal and plant protein intake were related to differential metabolomic profiles associated with BP. This study included 1008 adults from the African-PREDICT study (aged 20–30 years). Protein intake was determined using 24-h dietary recalls. Twenty-four-hour ambulatory BP was measured. Amino acids and acylcarnitines were analysed in spot urine samples using liquid chromatography-tandem mass spectrometry-based metabolomics. Participants with a low plant, high animal protein intake had higher SBP (by 3 mmHg, *p* = 0.011) than those with high plant, low animal protein intake (low-risk group). We found that the relationships of plant and animal protein intake with 24-h SBP were partially mediated by BMI and saturated fat intake, which were independently associated with SBP. Protein intake was therefore not related to SBP in multiple regression analysis after adjusting for confounders. In the low-risk group, methionine (Std. *β* = −0.217; *p* = 0.034), glutamic acid (Std. *β* = −0.220; *p* = 0.031), glycine (Std. *β* = −0.234; *p* = 0.025), and proline (Std. *β* = −0.266; *p* = 0.010) were inversely related to SBP, and beta-alanine (Std. *β* = −0.277; *p* = 0.020) to DBP. Ultimately a diet high in animal and low in plant protein intake may contribute to higher BP by means of increased BMI and saturated fat intake. Conversely, higher levels of urinary AAs observed in adults consuming a plant rich diet may contribute to lower BP.

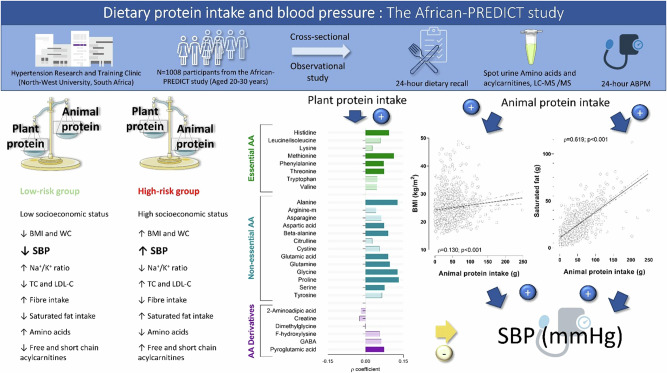

## Introduction

Dietary intervention is an established non-pharmacological strategy that plays an integral part in the prevention and management of hypertension [1], one of the largest public health concerns affecting over 1 billion adults globally [2]. The continued development of comprehensive dietary recommendations therefore remains a major priority. Although regional dietary patterns are linked with food availability, affordability, tradition, and culture, there is global consensus, aligning with WHO recommendations, with regard to what is considered elements of a healthy diet (e.g., increasing plant-based food intake and limiting saturated fat and sugar intake) [3, 4]. Metabolomics is a useful tool in nutrition science that may assist in the future development of more attainable and impactful dietary recommendations [5]. Continuing advances in the field of metabolomics have provided exciting new opportunities to gain better insights into metabolic pathways that may be involved in the link between nutrition and disease development [5, 6].

Dietary habits that contribute to increased cardiovascular risk include the inadequate consumption of plant- based proteins (whole grains, legumes, nuts, and seeds) [7, 8] as well as excessive animal protein intake, specifically red meat and processed meat [7]. In the different regions of Sub-Saharan Africa, low fruit, vegetable, whole grains, and legume consumption, together with high sodium intake, were the leading dietary risk factors for cardiovascular disease (CVD) death and disability-adjusted life years [7]. Although there are some discrepancies in the literature, the consumption of high-quality plant-based diets have often been associated with a decrease in blood pressure (BP) [9] and a lower risk of CVD mortality [10, 11]. Higher animal protein intake, on the other hand, is associated with a greater risk of hypertension [12] and CVD mortality [13]. The contrasting relationships of plant and animal protein intake with CVD risk may be attributed to the differential intrinsic amino acid (AA), fat, micronutrient, haem-iron, dietary fibre, and phytochemical compositions as well as their interactions with the gut microbiome [14, 15]. Using data from the USDA database [16], Lépine et al. [14] demonstrated that animal protein sources contain higher levels of essential amino acids (EAAs), including branched-chain amino acids (BCAAs) and aromatic amino acids, while plant protein sources are rich in nonessential AAs [14]. Evidently, higher levels of BCAAs [17, 18] and their short-chain acylcarnitine byproducts [19], as well as lower levels of certain nonessential AAs such as glycine [18, 20], serine [20], histidine [21] and others [22], have been associated with elevated BP and an increased risk of hypertension. This data suggests a link between the type of protein intake, the metabolome, and BP. However, ancillary research in different population groups is required to reinforce these findings. Corroborating evidence is needed to validate biomarkers that may be used to strengthen and support dietary recall data when assessing dietary risk.

The main aim of this study was to determine whether plant and animal protein intake may be differentially related to non-protein components (e.g., fibre, saturated fat or salt intake) or a metabolomic profile which in turn is associated with BP. We hypothesised that there will be a significant difference in the BP, body composition, non-protein component levels, and metabolomic profiles of participants consuming a low animal, high plant protein intake (low-risk dietary group) compared with those consuming a high animal, low plant protein intake (high-risk dietary group).

## Methods

### Study design and participant selection

The African Prospective study on the Early Detection and Identification of CVD and Hypertension (African-PREDICT) included 1202 young adults from communities in and around the Potchefstroom area in the North West Province of South Africa [23]. Inclusion criteria for the African-PREDICT study were 20–30 years of age, clinic BP < 140/90 mmHg during the screening phase, no self-reported chronic diseases or treatment thereof, HIV-uninfected, not pregnant or lactating. This study included *N* = 1008 participants from the African-PREDICT study with metabolomics and protein intake data. There was an equal sex (49.8% women and 50.2% men) and racial (49.9% Black and 50.1% White) distribution within the cohort.

All participants gave written informed consent prior to data collection. The African-PREDICT study complies with the Declaration of Helsinki, was approved by the Health Research Ethics Committee of the North-West University (NWU-00001-12-A1) and is registered on ClinicalTrials.gov (NCT03292094).

### Questionnaire, anthropometric, and physical activity data

Self-reported age, sex (male/female), race (Black/White) and socioeconomic status (SES) data were obtained from validated General Health and Demographic Questionnaires. Dietary protein, fibre, and fat intakes were determined by three 24-h dietary recall interviews (including 1 weekend day) using a standardised dietary collection kit (example pictures, packages, measurement tools, and food models) and the five-step multiple-pass approach. Participants’ body weight (kg) (SECA electronic scales, SECA, Birmingham, UK), height (m) (SECA stadiometer, SECA, Birmingham, UK) and waist circumference (WC, cm) (anthropometric nonflexible tape measure, Holtain, Ltd, Crymych, UK) were measured in triplicate, with the average of the three measurements used, and body mass index (BMI) calculated [(kg)/height (m^2^)]. Participants wore a triaxial accelerometer for seven days during which total energy expenditure (TEE) was measured as an estimate of participants’ physical activity (ActiHeart; CamNtech, Cambridge, UK), and corrected for body weight (TEE/kg/day).

### Ambulatory blood pressure monitoring

Ambulatory BP was measured in 30-min intervals during the day (06:00 to 22:00) and hourly at night (22:00 to 06:00). Participants were fitted with an appropriately sized brachial BP cuff on the non-dominant arm (Card(X)plore device, Meditech, Budapest, Hungary). In this study *N* = 979 participants had ≥12 successful day and ≥4 successful night inflations [24]. The mean successful inflation rate over the 24-h period was 88% with a standard deviation of 12.1%.

### Biological sampling and biochemical analyses

Participants fasted from 22:00 on the evening prior to the day of data collection. Early morning spot urine and blood samples were collected from participants and immediately taken to the onsite laboratory for preparation and storage (stored at −80 ^▫^C). Serum total cholesterol, high- (HDL-C) and low- (LDL-C) density lipoprotein cholesterol, gamma-glutamyl transferase (GGT) and C-reactive protein (CRP) as well as glycated haemoglobin (HbA1c) from EDTA whole blood were measured using the Cobas Integra 400plus (Roche, Basel Switzerland). Amino acids and acylcarnitines were measured from spot urine samples using liquid chromatography-tandem mass spectrometry (LC-MS/MS) (Agilent 6410 LC-MS/MS system with 1200 series LC front-end) as previously described [19]. We applied a 50% CV filter to the data, and metabolites with more than 50% zero values were removed from the data matrix. Twenty-four-hour urine samples were collected from participants according to WHO/PAHO guidelines [25] to measure urinary sodium and potassium (Cobas Integra 400plus, Roche, Basel Switzerland). Estimated salt intake was calculated.

$$	{Estimated\; salt\; intake}\left(g/{day}\right) \\ 	= \frac{{UNa}\left({mmol}/L\right)* {Uvolume}(L/24{hr})* 23\left({molecular\; weight\; Na}\right)}{390}$$

### Statistical analyses

All data analyses were performed using SPSS statistical software (IBM Corp. Released 2021. IBM SPSS Statistics for Windows, Version 28.0. Armonk, NY: IBM Corp). Data distributions in the total population were inspected using skewness, kurtosis, histograms, and Q-Q normality plots. We stratified participants into low- and high-risk groups according to dietary protein intakes. Firstly, we determined the tertiles of plant and animal protein intake within Black men, Black women, White men, and White women respectively, since consumption patterns differed between these groups. All participants within the lowest tertile of animal protein intake and highest tertile of plant protein intake from the respective sex and racial group stratifications were classified as being in the low-risk group. Conversely, all participants within the lowest tertile of plant protein intake and highest tertile of animal protein intake from the respective sex and racial group stratifications were classified as being in the high-risk group. Comparisons of SES, anthropometric, BP, biochemical, and dietary intake profiles between low- and high-risk dietary groups were performed using independent-T tests (normally distributed data) and Mann–Whitney U tests (skewed data including protein intake and metabolite data). Analysis of covariance (ANCOVA) was used to determine whether BP differed significantly between the low- and high-risk groups (adjusted for BMI, total cholesterol, LDL-C, salt intake, potassium excretion, Na^+^/K^+^ ratio, total energy intake, dietary fibre intake, or saturated fat intake). In the total group, we investigated the relationships of total, plant, and animal protein intake with 24-h BP using Spearman correlations and multiple linear regression analyses (pairwise deletion of data) adjusting for sex, race, SES, age, BMI, TEE, Na^+^/K^+^ ratio, LDL-C, dietary fibre intake, and saturated fat intake. Mediation analyses were performed to determine whether the relationship of plant and animal protein intake with 24-h SBP was mediated by any of the measured covariates. The associations of amino acid and acylcarnitine profiles with the different dietary protein intakes in the total cohort were determined using Spearman correlations (unadjusted and adjusted for sex, race, age, BMI, TEE, and total energy intake). We subsequently determined if AA and acylcarnitine levels differed between the low-risk and high-risk dietary protein intake groups using Mann–Whitney U test. ANCOVA was performed, adjusting for BMI and total protein intake, or BMI and total energy intake, to determine whether metabolomic differences between groups were confounded by these covariates. The matrix subcommand was used to compute partial correlations based on Spearman Rho’s. To lower the false discovery rate of statistically significant relationships we calculated the adjusted *p* values using the Benjamini-Hochberg procedure (reported as q-values). Lastly, we investigated the associations between 24-h BP and metabolites that were found to be significantly different between the low- and high-risk protein intake groups (Spearman correlations and multiple linear regression analyses adjusting for sex, race, age, BMI, TEE, and total energy intake). Although protein intake and metabolite data were skewed, P–P plots in multiple linear regression models were examined to ensure normal distribution of residuals as well as inspecting the homoscedasticity of the data. The main dependent variable, 24-h BP, was normally distributed.

## Results

The characteristics of this study cohort, aged 24 ± 3.12 years, are presented in Table 1. There was an equal sex and racial distribution between the low-risk (low animal, high plant protein intake) and high-risk (low plant, high animal protein intake) dietary groups. A greater proportion of the participants in the high-risk protein intake group were from a high SES (40% vs. 30% from low SES) while greater proportion of the low-risk protein intake group were from a low SES (45.5% vs. 25.7% from high SES).

**Table 1:** Characteristics of the study population

	Total group *N* = 1008	Low-risk group *N* = 102 (Low animal & high plant protein intake)	High-risk group *N* = 90 (Low plant and high animal protein intake)	Mean difference (SE)	*p* value
Men, *N* (%)	506 (50.2)	50 (49.0)	48 (53.3)		0.55
White ethnicity, *N* (%)	503 (49.9)	55 (53.9)	48 (53.3)		0.94
Socioeconomic status					0.049
Low	402 (39.9)	46 (45.5)	27 (30.0)		
Middle	303 (30.1)	29 (28.7)	27 (30.0)		
High	302 (30.0)	26 (25.7)	36 (40.0)		
Age (years)	24.5 ± 3.12	24.1 ± 3.23	24.7 ± 3.23		0.15
Anthropometric profile					
Waist circumference (cm)	78.5 (16.7)	75.1 (15.0)	83.9 (17.3)	6.73 (1.76)	<0.001
Body Mass Index (kg/m^2^)	24.2 (6.72)	22.3 (6.48)	25.9 (6.32)	2.90 (0.81)	<0.001
Physical activity					
TEE (kCal/day/kg)	32.6 ± 4.60	33.3 ± 4.81	32.3 ± 4.58	−1.08 (0.77)	0.16
Blood pressure profile					
24-h SBP (mmHg)	117 ± 9.42	114 ± 9.26	117 ± 8.45	3.32 (1.30)	0.011
24-h DBP (mmHg)	68 ± 5.77	67 ± 5.25	68 ± 5.62	1.17 (0.79)	0.14
Urinary profile					
Salt intake (g/day)	8.12 (5.77)	8.53 (6.21)	7.88 (6.23)	−0.35 (0.75)	0.77
Sodium excretion (mmol/day)	137.6 (97.8)	144.6 (105)	133.6 (105)	−5.90 (12.8)	0.77
Potassium excretion (mmol/day)	44.1 (37.8)	40.4 (26.4)	51.1 (43.3)	11.5 (4.45)	0.039
Na^+^/K^+^ ratio	3.21 (2.23)	3.36 (2.64)	2.79 (2.06)	−0.67 (0.29)	0.017
Biochemical profile					
Total cholesterol (mmol/L)	3.66 ± 1.16	3.36 ± 1.13	3.90 ± 1.18	0.54 (0.17)	0.002
HDL-C (mmol/L)	1.11 ± 0.40	1.03 ± 0.36	1.13 ± 0.45	0.10 (0.06)	0.098
LDL-C (mmol/L)	2.39 ± 0.93	2.16 ± 0.84	2.55 ± 0.90	0.39 (0.13)	0.003
HbA1c (%)	5.31 ± 0.32	5.32 ± 0.29	5.29 ± 0.34	−0.03 (0.05)	0.50
GGT (U/L)	17.1 (16.8)	15.1 (14.0)	17.8 (17.1)	4.94 (2.20)	0.007
CRP (mg/L)	0.83 (2.17)	0.54 (1.73)	1.12 (2.22)	0.45 (0.49)	0.007
24-h Dietary recall					
Energy intake (KJ)	7702 (3590)	8222 (3307)	7516 (2384)	−461.5 (326)	0.13
Total protein intake (g)	69.5 (40.0)	56.7 (24.1)	88.2 (35.2)	34.9 (3.26)	<0.001
Plant protein intake (g)	19.0 (12.2)	28.9 (10.5)	11.8 (4.49)	−20.4 (1.35)	<0.001
Animal protein intake (g)	41.2 (36.2)	19.8 (15.2)	74.2 (34.2)	57.4 (2.98)	<0.001
Potassium intake (mg)	1851 (1175)	1809 (921)	2116 (1121)	205 (111)	0.031
Fibre intake (g)	13.0 (9.46)	19.1 (11.0)	8.92 (4.77)	−11.3 (1.04)	<0.001
Total fat (g)	66.7 (46.0)	65.6 (47.8)	80.2 (50.9)	14.0 (5.06)	0.007
Saturated fat (g)	20.9 (16.5)	20.3 (14.1)	26.7 (21.1)	8.37 (1.75)	<0.001
Mono-unsaturated fat (g)	22.8 (17.7)	20.9 (17.2)	27.9 (19.4)	6.56 (1.89)	0.001
Polyunsaturated fat (g)	14.4 (12.9)	15.3 (16.1)	14.0 (10.8)	−1.84 (1.68)	0.51

Normally distributed data presented as arithmetic mean ± standard deviation. Compared using independent T-tests

Skewed data presented as median (IQR). Compared using Mann–Whitney U tests

Mean difference (SE) was determined from independent t-tests

*CRP* C-reactive protein, *DBP* Diastolic blood pressure, *GGT* Gamma-glutamyl transferase, *HDL-C* High-density lipoprotein cholesterol, *LDL-C* Low-density lipoprotein cholesterol, *SBP* Systolic blood pressure, *TEE* Total energy expenditure

### Relationship between dietary protein intake and 24-h blood pressure

Participants within the high-risk protein intake group had higher 24-h SBP, but not DBP, than participants in the low-risk protein intake group (*p* = 0.011). Considering possible confounding factors (BMI, total cholesterol, LDL-C, salt intake, potassium excretion, Na^+^/K^+^ ratio, total energy intake, dietary fibre intake, and saturated fat intake), the difference between groups became non-significant after adjustment for BMI (*p* = 0.19), LDL-C (*p* = 0.053), potassium excretion (*p* = 0.26), Na^+^/K^+^ ratio (*p* = 0.09), or saturated fat intake (*p* = 0.15) (Supplementary Table 1).

**Fig. 1:**
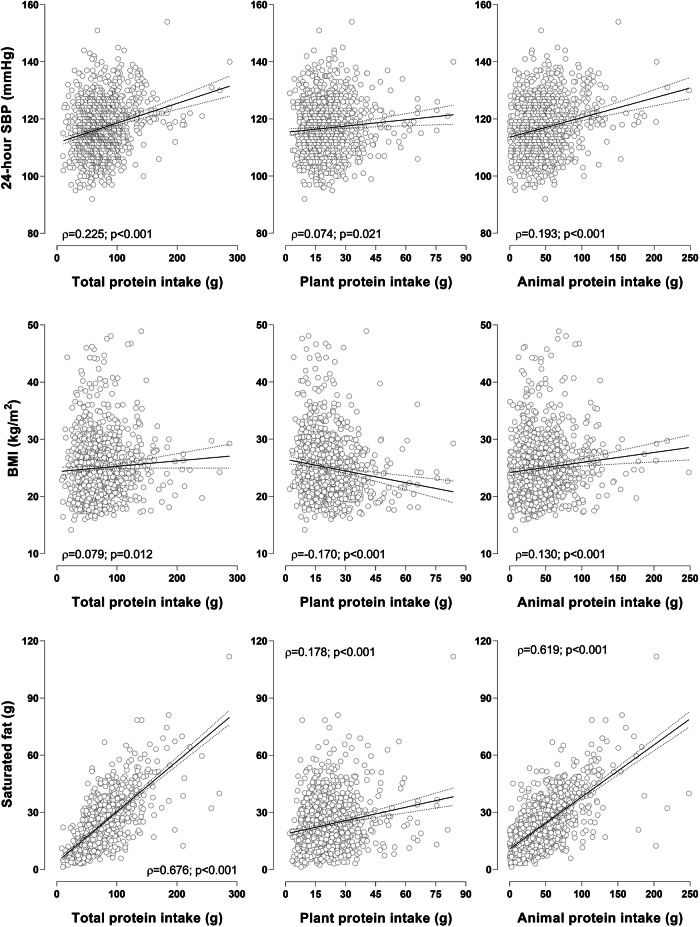
Spearman correlations of protein intake with 24-h SBP, BMI, and saturated fat intake in the total population

**Fig. 2:**
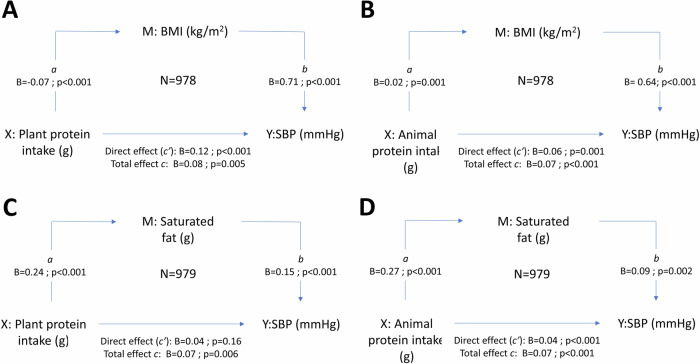
Mediating effect of BMI and saturated fat intake on the relationship of plant and animal protein intake with 24-hour systolic blood pressure. **A** Mediating effect of BMI on the relationship between plant protein intake and SBP; **B** Mediating effect of BMI on the relationship between animal protein intake and SBP; **C** Mediating effect of saturated fat intake on the relationship between plant protein intake and SBP; **D** Mediating effect of saturated fat intake on the relationship between animal protein intake and SBP. BMI Body mass index, M Mediator, SBP Systolic blood pressure, X independent variable, Y dependent variable

Although total (*ρ* = 0.225; *p* < 0.001), plant (*ρ* = 0.074; *p* = 0.021) and animal (*ρ* = 0.193; *p* < 0.001) protein intake correlated positively with SBP (Fig. 1), the relationships lost significance with backward stepwise multiple regression models (adjusting for sex, race, SES, age, BMI, TEE, Na^+^/K^+^ ratio, LDL-C, dietary fibre intake, and saturated fat intake). In the total cohort, male sex, BMI (adj. *R*^2^ = 0.44; Std. *β* = 0.44; *p* < 0.001) and saturated fat (adj. *R*^2^ = 0.44; Std. *β* = 0.082; *p* = 0.008) were significantly and positively associated with 24-h SBP. Performing mediation analyses in the total group (Fig. 2, Supplementary Table 2), the relationship between plant protein intake and SBP was partially mediated by BMI (mediation: −63.3%) (Fig. 2A) and fully mediated by saturated fat intake (mediation: 49.6%) (Fig. 2C). The relationship between animal protein intake and SBP was partially mediated by both BMI (mediation: 16.7%) (Fig. 2B) and saturated fat intake (mediation: 35.5%) (Fig. 2D). When performing backward stepwise multiple regression in the low and high-risk groups respectively, we found that male sex and BMI remained significantly associated with SBP in both groups.

### Relationship of dietary protein intake with urinary amino acids and acylcarnitines

**Fig. 3:**
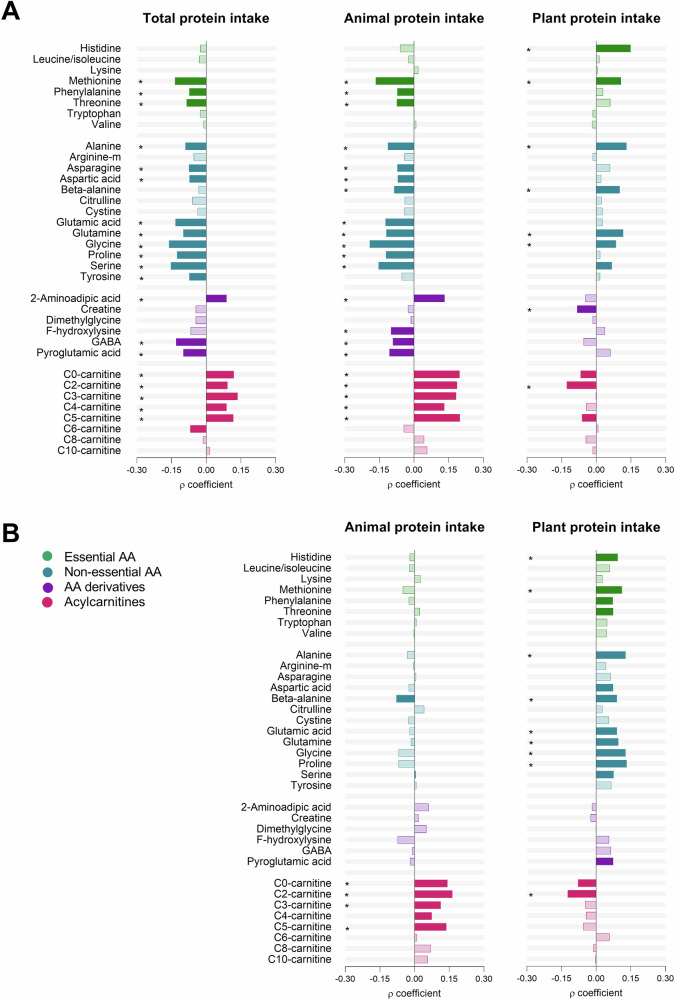
Relationships of protein intake with urinary amino acids and acylcarnitines. **A** Unadjusted Spearman correlations of total, animal, and plant protein intake with urinary amino acids and acylcarnitines. **B** Partial Spearman correlations of animal and plant protein intake with amino acids and acylcarnitines adjusted for sex, ethnicity, BMI, TEE, and total protein intake. Solid bars present correlations with significant *p* < 0.05. *Indicates significant *q* < 0.05

Spearman correlations revealed opposing relationships of animal and plant protein intake with amino acids and acylcarnitines (Fig. 3). After adjusting for sex, race, BMI, and total protein intake, animal protein intake correlated positively with C0 [free]-carnitine (*r* = 0.142) and short-chain acylcarnitines C2 [acetyl]- (*r* = 0.163), C3 [propionyl]- (*r* = 0.113) and C5 [isovaleryl]-carnitine (*r* = 0.137) (all *p* and *q* < 0.05). Conversely, plant protein intake correlated positively with histidine (*r* = 0.093), methionine (*r* = 0.112), alanine (*r* = 0.127), beta-alanine (*r* = 0.090), glutamic acid (*r* = 0.090), glutamine (*r* = 0.096), glycine (*r* = 0.127), and proline (*r* = 0.132), but negatively with C2-carnitine (*r* = −0.123) (all *p* and *q* < 0.05).

### Metabolite profiles of low animal, high plant protein intake (low-risk) vs. low plant, high animal protein intake (high-risk) groups

Comparisons of the metabolic profiles between the low-risk and high-risk dietary protein intake groups are shown in Supplementary Table 3. Participants within the high-risk group exhibited lower AA (EAAs: methionine and phenylalanine; non-essential AAs: alanine, aspartic acid, beta-alanine, glutamic acid, glutamine, glycine, proline, serine; AA derivatives: GABA and pyroglutamic acid) and C6 [hexanoyl] -carnitine levels, but higher C0-carnitine and short-chain acylcarnitines (C2- and C3-carnitine) levels (all *p* and *q* values < 0.05) compared to the low-risk group. After adjustment for BMI and total protein intake, which were significantly higher in the high-risk group, only beta-alanine levels remained significantly lower in the high-risk compared to the low-risk group (*q* < 0.001). Given the high collinearity of total protein intake and total energy intake (*r* = 0.731; *p* < 0.001), we did not adjust for both covariates simultaneously. After adjusting for BMI and total energy intake in sensitivity analyses, beta-alanine and glycine were significantly lower in the high-risk compared to the low-risk group (*q* = 0.018).

### Associations of 24-h blood pressure with urinary amino acids and acylcarnitines related to protein intake

**Fig. 4:**
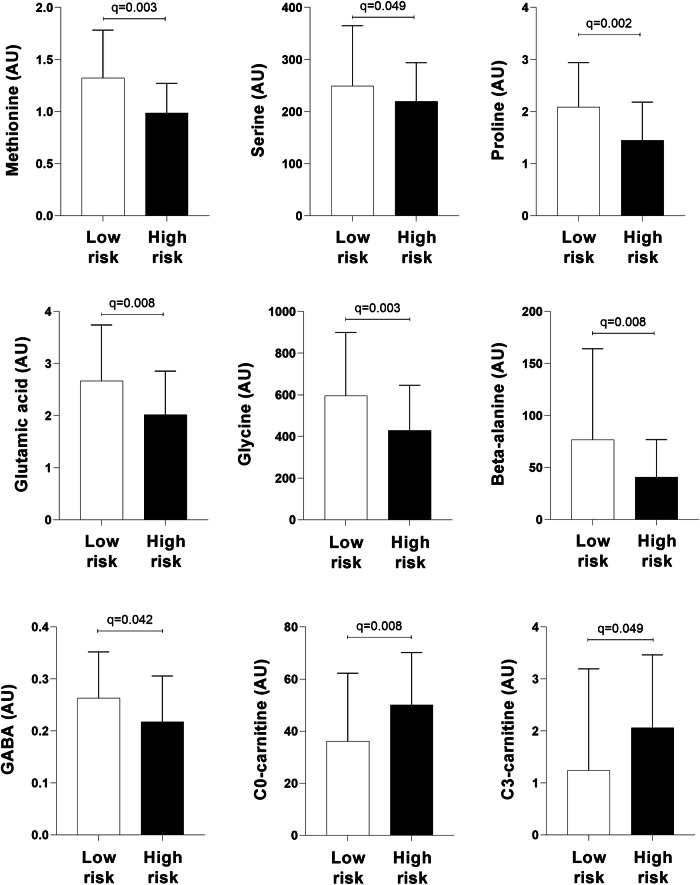
Comparison of metabolites that differed significantly between low-risk (low-animal-, high plant protein intake) vs. high-risk (low plant-, high animal protein intake) protein intake groups. Urinary metabolites are reported as arbitrary units (AU)

We subsequently determined whether metabolites that differed significantly between the high- and low-risk protein intake groups (Fig. 4) were associated with higher 24-h SBP. Analyses were adjusted for race, age, BMI, TEE, and total energy intake. In the total group, glycine (adj. *R*^2^ = 0.23; Std. *β* = −0.089; *p* = 0.005), GABA (adj. *R*^2^ = 0.23; Std. *β* = −0.103; *p* = 0.002), C0- (adj. *R*^2^ = 0.23; Std. *β* = 0.103; *p* = 0.001) and C3- (adj. *R*^2^ = 0.24; Std. *β* = 0.129; *p* < 0.001) levels associated with SBP (Table 2). Additionally, 24-h DBP inversely associated with beta-alanine (adj. *R*^2^ = 0.13; Std. *β* = −0.071; *p* = 0.036) and GABA (adj. *R*^2^ = 0.13; Std. *β* = −0.087; *p* = 0.015). All relationships lost significance with additional adjustment for sex. Stratified according to risk groups (Supplementary Table 4), we found that methionine (adj. *R*^2^ = 0.31; Std. *β* = −0.217; *p* = 0.034), glutamic acid (adj. *R*^2^ = 0.31; Std. *β* = −0.220; *p* = 0.031), glycine (adj. *R*^2^ = 0.31; Std. *β* = −0.234; *p* = 0.025), and proline (adj. *R*^2^ = 0.33; Std. *β* = −0.266; *p* = 0.010) associated inversely with SBP, and beta-alanine (adj. *R*^2^ = 0.15; Std. *β* = −0.277; *p* = 0.020) with DBP, in the low-risk protein intake group. The latter relationship between beta-alanine and DBP remained significant after additional adjustment for sex. In the high-risk group, serine associated inversely with SBP (adj. *R*^2^ = 0.23; Std. *β* = −0.223; *p* = 0.042).

**Table 2:** Relationship between 24-h blood pressure with metabolites that differed significantly between the low-risk (high plant, low animal protein intake) and high-risk (low plant, high animal protein intake) protein intake groups

	24-h SBP (mmHg)					
	Spearman correlation			Model 1		
	*ρ*	*p*	*q*	Adj. *R*^2^	Std. *β*	*p*
Methionine	−0.008	ns		0.22	−0.024	ns
Phenylalanine	0.085	0.008	0.019	0.22	−0.015	ns
Alanine	0.082	0.011	0.024	0.22	0.010	ns
Aspartic acid	−0.018	ns		0.22	−0.025	ns
Beta-alanine	0.004	ns		0.22	−0.050	ns
Glutamic acid	−0.045	ns		0.22	−0.029	ns
Glutamine	0.017	ns		0.22	−0.010	ns
Glycine	−0.065	0.041	0.076	0.23	−0.089	0.005
Proline	−0.097	0.002	0.008	0.22	−0.049	ns
Serine	−0.041	ns		0.22	−0.028	ns
GABA	−0.155	<0.001	<0.001	0.23	−0.103	0.002
Pyroglutamic acid	−0.018	ns		0.22	−0.021	ns
C0-carnitine	0.134	<0.001	<0.001	0.23	0.103	0.001
C2-carnitine	0.076	0.018	0.038	0.22	0.017	ns
C3-carnitine	0.163	<0.001	<0.001	0.24	0.129	<0.001
C6-carnitine	0.008	ns		0.22	−0.013	ns

	24-hour DBP (mmHg)					
	Spearman correlation			Model 1		
	*ρ*	*p*	*q*	Adj. *R*^2^	Std. *β*	*p*
Methionine	−0.020	ns		0.13	−0.044	ns
Phenylalanine	0.042	ns		0.13	−0.018	ns
Alanine	0.059	ns		0.13	0.039	ns
Aspartic acid	−0.015	ns		0.13	−0.032	ns
Beta-alanine	−0.046	ns		0.13	−0.071	0.036
Glutamic acid	−0.047	ns		0.13	−0.038	ns
Glutamine	−0.002	ns		0.13	−0.028	ns
Glycine	−0.035	ns		0.13	−0.060	ns
Proline	−0.077	0.017	ns	0.13	−0.036	ns
Serine	−0.023	ns		0.12	−0.016	ns
GABA	−0.127	<0.001	0.006	0.13	−0.087	0.015
Pyroglutamic acid	−0.036	ns		0.13	−0.030	ns
C0-carnitine	0.056	ns		0.13	0.036	ns
C2-carnitine	0.030	ns		0.12	0.000	ns
C3-carnitine	0.066	0.047	ns	0.13	0.047	ns
C6-carnitine	−0.012	ns		0.13	−0.034	ns

Model 1. Multiple linear regression adjusted ethnicity, age, BMI, TEE, and total energy intake

## Discussion

The main findings of this study included that (1) animal protein intake correlated positively with BMI and saturated fat intake which were the main determinants of higher SBP; (2) participants with a low animal, high plant protein intake (low-risk dietary group) had higher AAs and hexanoyl-carnitine, but lower free carnitine and short-chain acylcarnitine levels compared to the high-risk group; (3) in the low-risk group, higher levels of methionine, glutamic acid, glycine, proline and beta-alanine were related to BP. Ultimately, the means by which dietary protein intake contributes to BP may be attributed to the distinct protein characteristics.

Evidently, high animal protein intake [12], specifically red meat and processed meat [26–30], has been associated with higher BP and hypertension, while plant-based diets are associated with lower BP [9]. The contrasting relationships of plant and animal protein intake with CVD risk has been ascribed to the unique protein characteristics associated with different protein sources — including non-protein components (fibre, micronutrients, fats, carbohydrates, phytochemicals) and AA composition [14, 15, 31]. Plant-based protein sources typically contain less saturated fatty acids and more cardioprotective micronutrients, while animal protein sources are rich in saturated fats that contribute to an adverse cholesterol profile [15, 31]. In this study, participants with a high animal, low plant protein intake (high-risk) indeed had higher 24-h SBP compared to those with low animal, high plant protein intake (low-risk). However, the difference in SBP between the groups was mediated by BMI, LDL-C, potassium excretion (a proxy marker of potassium intake), and saturated fat intake—all of which were higher in the high-risk compared to the low-risk protein intake group. Unexpectedly, salt intake did not differ between the low-and high-risk protein intake groups and was not related to SBP. Although we found that the protein type itself was not an independent predictor for SBP when considering mediating factors, a diet high in animal protein intake likely increases SBP by increasing saturated fat intake and BMI.

While animal protein sources are reportedly comprised of higher EAAs and less non-essential AAs than plant protein sources [14], we found that total plant protein intake was positively associated with almost all AAs (EAAs, non-essential AAs, and AA derivatives) in the current study. It is possible that our observation may be attributed to specific animal and plant protein food sources consumed by the participants in this study population, however assessing the metabolic profiles with different animal and plant protein food sources was beyond the scope of this study and should therefore be investigated in the future. In the low-risk group, the AAs methionine, glutamic acid, glycine, proline, and beta-alanine were inversely related to BP. It is therefore likely that higher levels of these AAs observed in those with a plant rich diet may be protective against higher SBP. In the high-risk group, serine associated inversely with SBP. In the total study cohort, glycine, beta-alanine, GABA, free carnitine, and propionyl-carnitine were related to BP. Although it is beyond the scope of this study, sex had a confounding effect on the relationship between metabolites and BP. It is thus recommended that future studies investigate the role of sex on metabolomic pathways related to BP.

### Methionine

The EAA, methionine, is generally lower in plant-based compared with animal-based protein sources (1% vs. 2.5% of the total protein), although, concentrations may vary amongst plant-based food sources [32]. The methionine content of potato, hemp, corn, and brown rice, for instance, were shown to be higher than that of oats, soy, and wheat [32]. It is possible that higher methionine levels in the low-risk group of this study, consuming a plant rich diet, may be attributed to the specific plant-based food sources consumed by the participants in this group. Our observation that increased methionine was inversely associated with BP in adults consuming a plant-rich diet contrasts with evidence from animal studies that suggest that a high methionine diet may increase CVD risk by promoting oxidative stress, inflammation, and vascular matrix remodelling [33]. Further research is needed to determine whether methionine from plant- and animal-based food sources may be differentially related to BP.

### Glycine

Glycine is a conditional EAA and a major structural component of collagen [34]. Glycine is required to maintain healthy collagen turnover [34], and has anti-inflammatory and anti-oxidative functions [35]. In addition, glycine has been shown to promote endothelial nitric oxide synthase expression in aortic rings of Sprague-Dawley rats, which increases the bioavailability of vasodilatory nitric oxide (NO) [36]. Higher levels of glycine were previously observed in younger adults with decreased arterial stiffness [37], and has also been associated with lower central SBP [21]. In the total group, and particularly in the low-risk protein intake group, glycine was inversely related to 24-h SBP. In accordance with our findings, others have also reported an inverse relationship of glycine with BP and the risk of hypertension [20, 38–40].

### Serine and proline

Serine may be obtained through dietary intake (soybeans, nuts, eggs, lentils, meat, and fish), or endogenously synthesised from glycine or glucose [41]. With regard to proline, ref. [42] reported that animal protein intake decreased significantly across increasing tertiles of dietary proline intake, while plant protein intake increased. Interestingly, the authors further demonstrated that proline from cheese or legumes were associated with increased BP, while proline from red meat was associated with lower SBP [42]. In this study cohort we found that serine and proline were significantly higher in adults consuming a diet rich in plant protein. Serine and proline were inversely associated with SBP in the high-risk and low-risk groups, respectively. The inverse relationships of serine and proline with SBP agree with findings from the INTERMAP study [22]. Our research group has also previously demonstrated that serine and proline levels are higher in young adults with decreased arterial stiffness [37], and that serine is inversely associated with central SBP [21].

### Beta-alanine

Beta-alanine is a non-essential and non-proteinogenic AA, synthesised endogenously in the liver but may also be acquired through dietary intake of meat [43]. Beta-alanine is the rate limiting precursor of carnosine that exerts several protective roles in the vasculature [44]. In a recent review, ref. [44] outlined the physiological activities of carnosine (increased NO bioavailability, improved angiogenesis, inhibition of advanced glycation end products, anti-oxidative and anti-inflammatory) that impacts vascular health and thereby BP. Whether the inverse association between beta-alanine and DBP in this study (total group and low-risk group) may be mediated by the vascular effects of carnosine is unknown. It has been shown that increased DBP is strongly related to increased arterial stiffness [45], and that lower levels of beta-alanine is associated with increased arterial stiffness in children [46] and young adults [37] who exhibit early vascular aging.

### GABA and glutamic acid

Plant-based foods are rich sources of GABA and glutamic acid [47]. Potatoes and tomatoes in particular have been shown to contain high quantities of GABA, while cereals, nuts, and legumes contain higher quantities of glutamic acid [47]. Advances in food technology have encouraged the use of microorganisms during food processing that will increase the GABA content of food. Generally, GABA concentrations are therefore also elevated in fermented products [48]. We found that urinary GABA and glutamic acid levels were indeed higher in adults consuming a plant protein rich compared to those consuming an animal protein rich diet. GABA is an inhibitory neurotransmitter that influences arterial pressure by regulating sympathetic outflow from the paraventricular nucleus (PVN) [49]. It is thus likely that higher levels of GABA may lead to suppressed sympathetic vasomotor outflow by the PVN, and thereby decrease vasoconstriction. Although glutamate (glutamic acid) is an excitatory neurotransmitter, the excitatory effects thereof can be attenuated by NO [49]. Stamler et al. [22] suggests that the favourable effect of dietary glutamic acid on BP, also observed in the IMTERMAP study, may be attributed to glutamate being a precursor of arginine, which, when converted to nitric oxide, is a potent vasodilator [22]. Notably, glutamic acid levels were shown to be higher in young adults with decreased arterial stiffness compared to those with increased stiffness [37].

### Free carnitine

Dietary free carnitine is primarily acquired through animal protein sources but may also be synthesised endogenously from lysine and methionine [50]. In this study, we found that increased animal protein intake was associated with higher levels of urinary free carnitine (which increases in parallel with dietary free carnitine intake [50]). Dietary free carnitine is a main precursor for the gut microbiome-derived metabolite trimethylamine N-oxide (TMAO), which is associated with increased arterial stiffness and SBP [51]. Although TMAO was not determined in this study, we demonstrated that urinary free carnitine levels were associated with 24-h SBP, which is in accordance with previous findings reported by ref. [52]. Notably, it was further shown in the African-PREDICT study population that adults with greater arterial stiffness had higher levels of urinary free carnitine compared to those with less arterial stiffness [37].

### Propionyl-carnitine

Propionyl-carnitine is a short-chain acylcarnitine that is derived from propionyl-CoA [53]. Propionyl-CoA can be produced through various metabolic pathways including the catabolism of BCAAs, oxidation of odd chain fatty acids, and the oxidation of cholesterol side chains [53]. In this study, urinary propionyl-carnitine, together with free carnitine and acetyl-carnitine, were higher in the group with higher animal protein consumption. Since we found no association between animal protein intake and BCAAs, it is possible that higher urinary propionyl-carnitine levels may reflect sufficient BCAA catabolism. Higher levels of short-chain acylcarnitines, but not medium- or long-chain acyl-carnitines, could reflect increased fatty acid beta-oxidation in adults consuming high animal protein diets. It is well known that fatty acid beta-oxidation plays an essential role in cardiac contractility [54], but there is also evidence that fatty acid beta-oxidation is utilised for endothelial-dependent vasodilation [55]. Our research group has previously reported a positive association between urinary propionyl-carnitine levels and left ventricular mass index [56], while also demonstrating higher levels of propionyl-carnitine in adults with increased arterial stiffness [37]. It is likely that the role of propionyl-carnitine in cardiovascular energetics may contribute to the positive association that we observed between propionyl-carnitine and 24-hour SBP in this study.

## Future perspectives

This study demonstrates differential relationships of animal and plant protein intakes with AAs and acylcarnitines. Given the culturally diverse population, there may be significant value in investigating the metabolome of different food sources consumed by the different population groups in South Africa. Linking distinct metabolites, and thereby metabolic pathways, to specific food sources may assist in procuring more effective personalised approaches to dietary intervention. Additional health benefits may include the identification of metabolic pathways implicated in food allergies and gaining new perspectives on the medicinal properties of food.

### Strengths and limitations

To our knowledge this is the first study investigating the triadic relationship between protein intake (total, plant, and animal), metabolites (amino acids and acylcarnitines), and 24-h BP in a young cohort from Sub-Saharan Africa. All measurements were conducted under highly controlled conditions at a well-equipped research facility. This hypothesis generating study is limited by its cross-sectional design and causality can therefore not be inferred. Limitations of 24-h dietary recall data may include errors in a participant’s memory or possible misperceptions of their food portions. The inclusion of known biomarkers associated with dietary protein intake such as urinary nitrogen would have been beneficial and should be considered in follow-up studies. This study made use of a targeted metabolomics approach, and further studies using untargeted metabolomics may identify additional metabolite classes related to protein intake that were not included in our study.

## Conclusion

Our study suggests that a diet high in animal but low in plant protein intake may contribute to higher BP, while a plant rich diet may contribute to lower BP. Our findings support the notion that the differential relationships of protein type with BP is likely due to the unique protein characteristics (non-protein components and AA composition). Higher animal protein intake may be related to increased BMI and saturated fat intake, which are significant determinants of SBP. Furthermore, we demonstrated that adults with a plant protein rich diet had higher levels of urinary AAs together with lower free and short chain acylcarnitines compared to those with an animal protein rich diet. In the adults with a plant protein rich diet, AAs that were higher, inversely associated with SBP, suggesting a protective role of increased plant protein intake against higher BP.

## Supplementary information

Supplementary Tables
