# Supplementary material for: Urinary metabolomics signature of animal and plant protein intake and its association with 24-h blood pressure: the African-PREDICT study
Source: Hypertens Res. 2024 Jul 4;47(9):2456–70. doi: 10.1038/s41440-024-01767-8 (PMC11374704; doi:10.1038/s41440-024-01767-8)
Supplement: Supplementary file 1 — Supplementary Tables [file 41440_2024_1767_MOESM1_ESM.docx]

**Supplementary Tables**

**Urinary metabolomics signature of animal and plant protein intake and its association with 24-hour blood pressure: The African-PREDICT study**

Michél Strauss-Kruger^a,b^, Marlien Pieters^b,c^, Tertia van Zyl^b,c^, Ruan Kruger^a,b^, Adriaan Jacobs^a,b^, Esmé Jansen van Vuren^a,b^, Roan Louw^d^, Carina Mels^a,b^

^a.^Hypertension in Africa Research Team (HART), North-West University, Potchefstroom 2520, North-West Province, South Africa.

^b.^SAMRC Extramural Unit for Hypertension and Cardiovascular Disease, Faculty of Health Sciences, North-West University, Potchefstroom, South Africa

^c.^ Centre of Excellence for Nutrition (CEN); North-West University, Potchefstroom 2520, South Africa

^d.^ Human Metabolomics, North-West University, Potchefstroom, 2520, North-West Province, South Africa.

**Correspondence:** Catharina M. Mels, PhD

Tel: +27 18 299 1983; fax: +27 18 285 2432; e-mail: [carina.mels@nwu.ac.za](mailto:carina.mels@nwu.ac.za)

Postal address: Hypertension in Africa Research Team (HART), Private Bag X6001, North-West University, Potchefstroom 2520, North-West Province, South Africa

**Supplementary Table 1.** Comparison of 24-hour SBP between low animal, high plant protein intake (low-risk) vs. low plant, high animal protein intake (high-risk) groups, adjusting for possible confounders.

|  | Low-risk group N=102  (Low animal & high plant protein intake) | High-risk group N=90  (Low plant and high animal protein intake) | Mean difference (SE) | p value |
| --- | --- | --- | --- | --- |
| Unadjusted |  |  |  |  |
| 24-hour SBP (mmHg) | 114 ± 9.26 | 117 ± 8.45 | 3.32 (1.30) | 0.011 |
| Adjusted for |  |  |  |  |
| BMI | 115 (113; 117) | 117 (115; 118) | 1.64 (-0.79; 4.08) | 0.19 |
| Total cholesterol | 114 (112; 116) | 117 (115; 119) | 2.84 (0.18; 5.49) | 0.036 |
| LDL-C | 114 (113; 116) | 117 (115; 119) | 2.59 (-0.03; 5.21) | 0.053 |
| Salt intake | 114 (113; 116) | 117 (115; 119) | 2.86 (0.19; 5.53) | 0.036 |
| Potassium excretion | 115 (113; 117) | 116 (114; 118) | 1.61 (-1.18; 4.39) | 0.26 |
| Na^+^/K^+^ ratio | 114 (113; 116) | 117 (115; 119) | 2.45 (-0.38; 5.28) | 0.090 |
| Total energy intake | 114 (112; 115) | 118 (116; 119) | 3.91 (1.45; 6.36) | 0.002 |
| Fibre intake | 114 (112; 116) | 118 (116; 120) | 4.31 (1.09; 7.53) | 0.009 |
| Saturated fat intake | 115 (113; 116) | 117 (115; 119) | 1.94 (-0.72; 4.60) | 0.15 |
| Comparisons made using ANCOVA.  BMI: Body mass index; LDL-C: Low density lipoprotein; SBP: Systolic blood pressure | | | | |

**Supplementary Table 2:** Mediation analyses

|  | **Effect** | **B** | **SE (B)** | **β** | **t** | **p** |
| --- | --- | --- | --- | --- | --- | --- |
| Mediation analyses where M: BMI; X: Plant protein intake; Y: 24-hour SBP | | | | | | |
| *a* | Plant protein intake -> BMI | -0.07 | 0.02 | -0.14 | -4.35 | <0.001 |
| *b* | BMI -> SBP | 0.71 | 0.05 | 0.41 | 14.1 | <0.001 |
| *c (Total)* | Plant protein intake -> SBP | 0.08 | 0.03 | 0.09 | 2.84 | 0.005 |
| *c’ (Direct)* | Plant protein intake -> SBP | 0.12 | 0.02 | 0.15 | 5.03 | <0.001 |
| *ab (Indirect)* | Plant protein intake -> BMI -> SBP | -0.05 (-0.07; -0.03) | | -0.06 (-0.08; -0.03) | |  |
|  | % mediation |  |  | -63.3 |  |  |
| Mediation analyses where M: BMI; X: Animal protein intake; Y: 24-hour SBP | | | | | | |
| *a* | Animal protein intake -> BMI | 0.02 | 0.01 | 0.11 | 3.31 | 0.001 |
| *b* | BMI -> SBP | 0.64 | 0.05 | 0.37 | 12.9 | <0.001 |
| *c (Total)* | Animal protein intake -> SBP | 0.07 | 0.01 | 0.24 | 7.58 | <0.001 |
| *c’ (Direct)* | Animal protein intake -> SBP | 0.06 | 0.05 | 0.20 | 12.9 | <0.001 |
| *ab (Indirect)* | Animal protein intake -> BMI -> SBP | 0.01 (0.005; 0.02) | | 0.04 (0.02; 0.06) | |  |
|  | % mediation |  |  | 16.7 |  |  |
| Mediation analyses where M: Saturated fat intake; X: Plant protein intake; Y: 24-hour SBP | | | | | | |
| *a* | Plant protein intake -> saturated fat intake | 0.24 | 0.04 | 0.20 | 6.42 | <0.001 |
| *b* | Saturated fat intake -> SBP | 0.15 | 0.02 | 0.22 | 6.81 | <0.001 |
| *c (Total)* | Plant protein intake -> SBP | 0.07 | 0.03 | 0.09 | 2.76 | 0.006 |
| *c’ (Direct)* | Plant protein intake -> SBP | 0.04 | 0.03 | 0.04 | 1.39 | 0.16 |
| *ab (Indirect)* | Plant protein intake -> saturated fat intake -> SBP | 0.04 (0.02; 0.06) | | 0.04 (0.02; 0.07) | |  |
|  | % mediation |  |  | 49.6 |  |  |
| Mediation analyses where M: Saturated fat intake; X: Animal protein intake; Y: 24-hour SBP | | | | | | |
| *a* | Animal protein intake -> Saturated fat | 0.27 | 0.01 | 0.65 | 27.0 | <0.001 |
| *b* | Saturated fat -> SBP | 0.09 | 0.03 | 0.13 | 3.11 | 0.002 |
| *c (Total)* | Animal protein intake -> SBP | 0.07 | 0.01 | 0.23 | 7.52 | <0.001 |
| *c’ (Direct)* | Animal protein intake -> SBP | 0.04 | 0.01 | 0.15 | 3.69 | <0.001 |
| *ab (Indirect)* | Animal protein intake -> saturated fat intake -> SBP | 0.02 (0.01; 0.04) | | 0.08 (0.03; 0.14) | |  |
|  | % mediation |  |  | 35.5 |  |  |
| For the indirect effect (*ab*) PROCESS estimated CI by bootstrapping. If the 95% CI does not contain 0 the p value is considered significant.  % mediation is calculated as the standardised indirect effect/ standardised total effect*100  BMI: Body mass index; M: Mediator; SBP: Systolic blood pressure; X: Independent variable; Y: Dependent variable | | | | | | |

**Supplementary Table 3.** Comparison of urinary metabolites between low animal, high plant protein intake (low-risk) vs. low plant, high animal protein intake (high-risk) groups.

|  | Low-risk group N=102 | High-risk group N=90 | Unadjusted | |  | Adjusted for BMI and total protein intake | |  | Adjusted for BMI and total energy intake | |
| --- | --- | --- | --- | --- | --- | --- | --- | --- | --- | --- |
|  | Low animal & high plant protein intake | Low plant and high animal protein intake | p | q |  | p | q |  | p | q |
| Histidine | 818.9 (741) | 635.3 (507) | **0.029** | ns |  | **0.029** | ns |  | **0.014** | ns |
| Leucine/isoleucine | 13.5 (7.07) | 12.9 (7.67) | 0.13 | ns |  | 0.47 | ns |  | 0.19 | ns |
| Lysine | 31.6 (36.2) | 31.7 (33.6) | 0.69 | ns |  | 0.83 | ns |  | 0.88 | ns |
| Methionine | 1.32 (0.82) | 0.99 (0.53) | **<0.001** | **0.003** |  | 0.20 | ns |  | **0.021** | ns |
| Phenylalanine | 19.1 (10.2) | 15.3 (10.6) | **0.010** | **0.031** |  | 0.16 | ns |  | 0.080 | ns |
| Threonine | 87.2 (76.6) | 75.7 (58.2) | 0.073 | ns |  | 0.43 | ns |  | 0.055 | ns |
| Tryptophan | 1.48 (1.16) | 1.14 (1.00) | 0.090 | ns |  | 0.62 | ns |  | 0.13 | ns |
| Valine | 5.27 (2.74) | 4.69 (2.81) | 0.14 | ns |  | 0.47 | ns |  | 0.25 | ns |
| Alanine | 265.6 (244) | 224.8 (158) | **0.005** | **0.021** |  | 0.13 | ns |  | **0.013** | ns |
| Arginine | 11.7 (7.06) | 11.0 (6.66) | 0.35 | ns |  | 0.43 | ns |  | 0.27 | ns |
| Asparagine | 24.2 (17.8) | 23.2 (18.7) | 0.13 | ns |  | 0.13 | ns |  | 0.066 | ns |
| Aspartic acid | 4.95 (3.14) | 4.21 (2.65) | **0.004** | **0.018** |  | 0.052 | ns |  | **0.039** | ns |
| Β-alanine | 76.6 (140) | 40.7 (62.1) | **0.001** | **0.008** |  | **<0.001** | **<0.001** |  | **<0.001** | **0.018** |
| Citrulline | 4.58 (3.99) | 4.31 (3.50) | 0.50 | ns |  | 0.70 | ns |  | 0.25 | ns |
| Cystine | 0.66 (0.50) | 0.51 (0.41) | 0.093 | ns |  | 0.34 | ns |  | **0.046** | ns |
| Glutamic acid | 2.67 (1.95) | 2.02 (1.39) | **0.002** | **0.008** |  | 0.40 | ns |  | **0.043** | ns |
| Glutamine | 268.5 (207) | 211.5 (167) | **0.008** | **0.029** |  | 0.29 | ns |  | **0.016** | ns |
| Glycine | 594.9 (489) | 427.7 (380) | **<0.001** | **0.003** |  | 0.051 | ns |  | **<0.001** | **0.018** |
| Proline | 2.09 (1.42) | 1.45 (1.08) | **<0.001** | **0.002** |  | 0.14 | ns |  | **0.009** | ns |
| Serine | 249.0 (181) | 219.8 (137) | **0.022** | **0.049** |  | 0.28 | ns |  | **0.010** | ns |
| Tyrosine | 37.8 (23.8) | 32.7 (20.8) | 0.063 | ns |  | 0.50 | ns |  | 0.079 | ns |
| 2-aminoadipic acid | 0.84 (0.78) | 0.89 (0.88) | 0.44 | ns |  | 0.53 | ns |  | 0.81 | ns |
| Creatine | 15.2 (10.8) | 14.9 (14.6) | 0.88 | ns |  | 0.89 | ns |  | 0.89 | ns |
| Dimethylglycine | 11.9 (15.6) | 10.5 (44.4) | 0.81 | ns |  | 0.68 | ns |  | 0.55 | ns |
| F hydroxylysine | 11.9 (9.11) | 10.5 (8.72) | **0.043** | ns |  | **0.015** | ns |  | **0.020** | ns |
| GABA | 0.26 (0.16) | 0.22 (0.14) | **0.015** | **0.042** |  | 0.63 | ns |  | 0.075 | ns |
| Pyroglutamic acid | 26.6 (16.8) | 21.8 (12.1) | **0.019** | **0.046** |  | 0.17 | ns |  | **0.027** | ns |
| C0-carnitine | 36.1 (40.0) | 50.0 (38.3) | **0.002** | **0.008** |  | 0.65 | ns |  | 0.15 | ns |
| C2-carnitine | 8.19 (18.7) | 16.4 (23.7) | **<0.001** | **0.003** |  | 0.88 | ns |  | 0.73 | ns |
| C3-carnitine | 1.24 (2.63) | 2.06 (2.42) | **0.021** | **0.049** |  | 0.95 | ns |  | 0.35 | ns |
| C4-carnitine | 9.12 (10.9) | 11.3 (14.9) | 0.079 | ns |  | 0.90 | ns |  | 0.32 | ns |
| C5-carnitine | 0.50 (0.58) | 0.61 (0.53) | **0.036** | ns |  | 0.84 | ns |  | 0.26 | ns |
| C6-carnitine | 0.06 (0.07) | 0.05 (0.04) | **0.014** | **0.041** |  | 0.20 | ns |  | **0.031** | ns |
| C8-carnitine | 0.30 (0.20) | 0.29 (0.17) | 0.73 | ns |  | 0.27 | ns |  | 0.31 | ns |
| C10-carnitine | 0.21 (0.18) | 0.18 (0.18) | 0.56 | ns |  | 0.27 | ns |  | 0.42 | ns |
| Data presented as median (IQR). Compared using Mann-Whitney U tests.  ANCOVA was performed to compare metabolites between groups while adjusting for BMI and total energy intake. | | | | | | | | | | |

**Supplementary Table 4.** Relationship between 24-hour blood pressure with metabolites that differed significantly between the low-risk (high plant, low animal protein intake) and high-risk (low plant, high animal protein intake) protein intake groups.

|  | **24-hour SBP (mmHg)** | | | | | | | | | | | | | | |
| --- | --- | --- | --- | --- | --- | --- | --- | --- | --- | --- | --- | --- | --- | --- | --- |
|  | **Low animal and high plant protein intake (Low-risk group)** | | | | | | |  | **Low plant and high animal protein intake (High-risk group)** | | | | | | |
|  | Spearman correlation | | |  | Model 1 | | |  | Spearman correlation | | |  | Model 1 | | |
|  | ρ | p | q |  | Adj. R^2^ | Std. β | p |  | ρ | p | q |  | Adj. R^2^ | Std. β | p |
| Methionine | -0.069 | 0.50 | ns |  | **0.31** | **-0.217** | **0.034** |  | -0.085 | 0.43 | ns |  | 0.20 | -0.123 | ns |
| Phenylalanine | 0.090 | 0.37 | ns |  | 0.26 | -0.076 | ns |  | -0.057 | 0.60 | ns |  | 0.19 | -0.088 | ns |
| Alanine | 0.160 | 0.12 | ns |  | 0.27 | -0.110 | ns |  | -0.070 | 0.52 | ns |  | 0.19 | -0.059 | ns |
| Aspartic acid | -0.092 | 0.36 | ns |  | 0.27 | -0.124 | ns |  | -0.037 | 0.73 | ns |  | 0.19 | -0.087 | ns |
| Beta-alanine | 0.102 | 0.32 | ns |  | 0.28 | -0.144 | ns |  | <0.001 | 0.99 | ns |  | 0.19 | -0.077 | ns |
| Glutamic acid | **-0.212** | **0.035** | ns |  | **0.31** | **-0.220** | **0.031** |  | -0.168 | 0.12 | ns |  | 0.20 | -0.124 | ns |
| Glutamine | 0.057 | 0.58 | ns |  | 0.30 | -0.191 | ns |  | **-0.217** | **0.042** | ns |  | 0.20 | -0.110 | ns |
| Glycine | -0.088 | 0.39 | ns |  | **0.31** | **-0.234** | **0.025** |  | -0.184 | 0.086 | ns |  | 0.21 | -0.178 | ns |
| Proline | **-0.216** | **0.032** | ns |  | **0.33** | **-0.266** | **0.010** |  | -0.091 | 0.40 | ns |  | 0.19 | -0.098 | ns |
| Serine | -0.019 | 0.85 | ns |  | 0.28 | -0.142 | ns |  | **-0.253** | **0.017** | ns |  | **0.23** | **-0.223** | **0.042** |
| GABA | **-0.241** | **0.021** | ns |  | 0.30 | -0.204 | 0.056 |  | -0.112 | 0.33 | ns |  | 0.19 | -0.123 | ns |
| Pyroglutamic acid | -0.052 | 0.61 | ns |  | 0.29 | -0.172 | ns |  | -0.053 | 0.63 | ns |  | 0.19 | -0.085 | ns |
| C0-carnitine | 0.100 | 0.33 | ns |  | 0.26 | 0.042 | ns |  | -0.035 | 0.75 | ns |  | 0.18 | 0.011 | ns |
| C2-carnitine | 0.125 | 0.22 | ns |  | 0.26 | -0.008 | ns |  | -0.140 | 0.19 | ns |  | 0.19 | -0.093 | ns |
| C3-carnitine | 0.122 | 0.28 | ns |  | 0.24 | 0.029 | ns |  | -0.095 | 0.39 | ns |  | 0.19 | -0.065 | ns |
| C6-carnitine | 0.150 | 0.14 | ns |  | 0.26 | 0.045 | ns |  | **-0.255** | **0.018** | ns |  | 0.22 | -0.176 | ns |
|  | **24-hour DBP (mmHg)** | | | | | | | | | | | | | | |
|  | **Low animal and high plant protein intake (Low-risk group)** | | | | | | |  | **Low plant and high animal protein intake (High-risk group)** | | | | | | |
|  | Spearman correlation | | |  | Model 1 | | |  | Spearman correlation | | |  | Model 1 | | |
| Methionine | -0.011 | 0.92 | ns |  | 0.09 | -0.118 | ns |  | 0.050 | 0.64 | ns |  | 0.11 | -0.046 | ns |
| Phenylalanine | 0.023 | 0.82 | ns |  | 0.08 | 0.037 | ns |  | 0.004 | 0.97 | ns |  | 0.11 | -0.063 | ns |
| Alanine | 0.081 | 0.43 | ns |  | 0.07 | -0.014 | ns |  | 0.085 | 0.98 | ns |  | 0.11 | 0.045 | ns |
| Aspartic acid | -0.027 | 0.79 | ns |  | 0.07 | 0.011 | ns |  | 0.076 | 0.48 | ns |  | 0.11 | 0.008 | ns |
| Beta-alanine | -0.118 | 0.25 | ns |  | **0.15** | **-0.277** | **0.020** |  | 0.118 | 0.28 | ns |  | 0.11 | 0.052 | ns |
| Glutamic acid | -0.151 | 0.14 | ns |  | 0.08 | -0.085 | ns |  | -0.065 | 0.55 | ns |  | 0.11 | -0.055 | ns |
| Glutamine | 0.072 | 0.48 | ns |  | 0.08 | -0.045 | ns |  | 0.011 | 0.92 | ns |  | 0.11 | -0.044 | ns |
| Glycine | -0.052 | 0.61 | ns |  | 0.09 | -0.112 | ns |  | 0.058 | 0.59 | ns |  | 0.11 | -0.003 | ns |
| Proline | **-0.245** | **0.015** | ns |  | 0.09 | -0.143 | ns |  | 0.040 | 0.71 | ns |  | 0.11 | -0.008 | ns |
| Serine | 0.029 | 0.78 | ns |  | 0.07 | -0.001 | ns |  | -0.056 | 0.60 | ns |  | 0.12 | -0.113 | ns |
| GABA | **-0.278** | **0.008** | ns |  | 0.10 | -0.172 | ns |  | -0.002 | 0.98 | ns |  | 0.09 | -0.046 | ns |
| Pyroglutamic acid | -0.080 | 0.43 | ns |  | 0.09 | -0.107 | ns |  | 0.035 | 0.75 | ns |  | 0.11 | -0.062 | ns |
| C0-carnitine | 0.146 | 0.15 | ns |  | 0.09 | 0.105 | ns |  | -0.181 | 0.091 | ns |  | 0.12 | -0.123 | ns |
| C2-carnitine | 0.195 | 0.052 | ns |  | 0.07 | 0.017 | ns |  | -0.079 | 0.46 | ns |  | 0.11 | -0.032 | ns |
| C3-carnitine | 0.087 | 0.44 | ns |  | 0.06 | 0.074 | ns |  | **-0.231** | **0.036** | ns |  | 0.11 | -0.096 | ns |
| C6-carnitine | -0.024 | 0.82 | ns |  | 0.07 | 0.004 | ns |  | -0.110 | 0.31 | ns |  | 0.13 | -0.151 | ns |
| **Model 1**. Multiple linear regression adjusted ethnicity, age, BMI, TEE, and total energy intake | | | | | | | | | | | | | | | |
